# Efficacy of cyclin-dependent kinase 4/6 inhibitors in patients with metastatic hormone positive breast cancer: a single institutional study from India

**DOI:** 10.3332/ecancer.2022.1450

**Published:** 2022-09-26

**Authors:** Sandip Ganguly, Navonil Mukherjee, Sayan Mandal, Somnath Roy, Sanjit Agarwal, Bivas Biswas, Joydeep Ghosh

**Affiliations:** 1 Department of Medical Oncology, Tata Medical Center, 14 Mar EW Arterial Road, Newtown, Kolkata 7000160, India; 2 Department of Breast Surgery, Tata Medical Center, 14 Mar EW Arterial Road, Newtown, Kolkata 7000160, India

**Keywords:** palbociclib, ribociclib, breast cancer, India

## Abstract

**Purpose:**

Cyclin-dependent kinase 4/6 (CDK4/6) inhibitors have shown marked benefit in the treatment of hormone positive metastatic breast cancer (HR+ MBC). There are limited real-world studies with palbociclib and ribociclib. Here we report our experience with CDK4/6 inhibitors in these groups of patients.

**Material and methods:**

Patients with HR+ MBC who have received either palbociclib or ribociclib during the course of their treatment from January 2017 to January 2022 were included in the study. The baseline demographic features, treatment details and toxicity were recorded. Patients who received at least 1 month of therapy were included in the survival analysis.

**Results:**

A total of 144 patients received CDK4/6 inhibitors during the time period. The median age of the population was 53 (30–80) years. Ninety-eight (71.4%) patients presented with *de novo* metastatic disease. The most common site of metastasis was to the skeleton (74.2%). Most patients (75%) received palbociclib as their therapy. At a median follow-up of 20.2 months, the median progression free survival (PFS) of the whole population was 16.5 (95% confidence interval (95% CI): 11.6–25.5) months and the median overall survival (OS) was 29.7 (95% CI: 21.7–44.6) months. The presence of liver metastases, low progesterone receptor positivity (Allred score < 6) and prior systemic treatment were poor prognostic factors for both PFS and OS in multivariate analysis. Drug was discontinued for only 2.1% of the patient population.

**Conclusions:**

Use of CDK4/6 inhibitors has led to improvement in PFS and OS in patients with HR+ MBC and it is well tolerated. The presence of liver metastases and low progesterone receptor positivity (Allred score < 6) and prior treatment are poor prognostic factors.

## Introduction

The treatment paradigm of hormone-positive metastatic breast cancer (HR+ MBC)/HER 2 negative metastatic breast cancer changed after approval of cyclin-dependent kinase 4/6 (CDK4/6). The use of CDK4/6 inhibitors has led to a marked improvement in disease control and progression-free survival (PFS) in both first-line and second-line settings [1]. For a long time, we had only two CDK4/6 inhibitors, palbociclib and ribociclib, in India, and recently abemaciclib has been made available. The present study describes the outcome of using both palbociclib and ribociclib in patients with HR+ MBC from India.

## Material and methods

Our study is a single institutional retrospective study of patients with HR+ MBC registered in the Department of Medical Oncology of the hospital. Patients who received CDK4/6 inhibitors during any line of treatment between January 2017 and January 2022 were included in the study. The institutional review committee granted a waiver for informed consent as this is a retrospective study.

### Diagnosis

All patients underwent tissue diagnosis by biopsy from the most accessible site followed by appropriate immunohistochemistry (IHC) markers. Hormone positivity was defined by oestrogen receptor (ER) positivity of more than 3 by Allred scoring system with any progesterone receptor (Pr) score [2]. Patients with an Allred score of 6 or more were considered to have strong hormonal positivity. The proliferative index was measured by using the MIB 1(Ki67 receptor) IHC. HER 2 positive patients were not included in the study. Computed tomography of thorax and abdomen with contrast along with bone scan or whole body 18F-fluorodeoxyglucose positron emission tomography-computed tomography was the modality used to stage the metastatic disease and to monitor response to the treatment.

### After diagnosis and treatment

Patients with *de novo* metastatic disease and those whose disease have recurred more than 12 months after completion of adjuvant therapy received either palbociclib (125 mg per day orally D1-21 Q28 days) or ribociclib (600 mg twice a day orally D1-21 Q28 days) along with letrozole 2.5 mg once daily. For a premenopausal female with HR+ MBC, hormonal therapy can be with either only tamoxifen/tamoxifen with ovarian function suppression (OFS)/aromatase inhibitors (AI) with OFS. In India, the companies have a patient assistance programme, where they provide the drug at free of cost after a fixed number of cycles when prescribed with either letrozole or fulvestrant. Because of the patient assistance programme, we have opted for the usage of OFS with AI along with the CDK4 inhibitors and did not opt for tamoxifen or tamoxifen with OFS. OFS was induced either by surgical or medical means before initiating CDK4/6 inhibitors for premenopausal females. Patients who have received prior hormonal therapy for advanced disease or progressed within 12 months of adjuvant hormonal treatment were also included in the study. They received CDK4/6 inhibitors with fulvestrant. Fulvestrant was administered intramuscularly at 500 mg on day 0, day 15 and day 28 and then monthly once. During the initial days of drug availability in the country, some patients have received either of the CDK4/6 inhibitors with any hormonal therapy after failure of two or more prior lines of treatment. The toxicity to the therapy was monitored, and it was graded as per Common Terminology Criteria for Adverse Events Version 5.0 [3]. The dose modification was done in case of toxicities, and it was permanently discontinued when patients had toxicity despite appropiate dose modifications or grade 4 toxicity. Complete blood count (CBC) was used to look for haematological toxicity. CBC was monitored 2-weekly during the first two cycles and then periodically based on the time of follow-up. Those who were coming monthly it was done monthly and for patients who were called for follow-up every 3 months it was done accordingly. Patients were assessed with conventional imaging to look for disease response. In a real-world setting, because of cost constraints, time interval between imaging was not uniform for all patients. For most it was initially done at the completion of 5 months of therapy and then periodically every 6 months. If there was symptomatic deterioration it was done earlier. Complete response (CR), partial response (PR), stable disease (SD) and progressive disease (PD) were defined as per Response Evaluation Criteria in Solid Tumours Version 1.1 [4].

### Statistical analysis

We have used descriptive statistics to analyse demographic details, clinical characteristics and treatment-related variables. We used the Kaplan Meier method to determine survival, and survival estimates were compared through the log-rank test. Data were censored on 31 March 2022. Censoring was done for the patients lost to follow-up or contact on the censoring date. PFS was estimated from the date of initiation of CDK4/6 inhibitors to the date of disease progression. Overall survival (OS) was calculated from the date of initiation of therapy to the date of death from either the disease or any cause. The Cox proportional hazard model was initially used to detect the outcome difference in the univariate analysis. We have included factors with significance (*p* < 0.05) in the univariate analysis for multivariate analysis. We have included treatment abandonment in the survival analysis of this study. It has been proposed that patients who do not comply with treatment and abandon it be included in survival analysis for studies from developing nations to provide an accurate picture of outcomes from these countries [5]. We have used STATA/SE 11.0 (StataCorp, College Station, Texas, USA) for data analysis.

## Results

A total of 144 patients received CDK4/6 inhibitors during our study period. The baseline characteristics of patients have been shown in Table 1. The median age of the study population was 53 (30–80 years). Twenty-seven (18%) patients were premenopausal at the time of initial diagnosis. Ninety-eight (71.4%) patients presented with *de novo* metastatic disease. Skeletal metastases were the most common site of metastases seen in 74.2% of cases. Among visceral metastases, the lung was the most common site of metastases, and it was seen in 71.4% of cases. The most common histology was invasive ductal carcinoma (92%). The treatment details and response rate of our study are shown in Table 2. In our study, most patients received palbociclib (80%). A total of 80% of the patients received CDK4/6 as their initial therapy for metastatic disease. Most of the patients received CDK4/6 inhibitors in combination with aromatase inhibitors. At present, 124 patients have been evaluated for response assessment. Among evaluable patients, the overall response rate was seen in 61% of patients, and the clinical benefit rate was seen in 79%.

**Figure 1.**
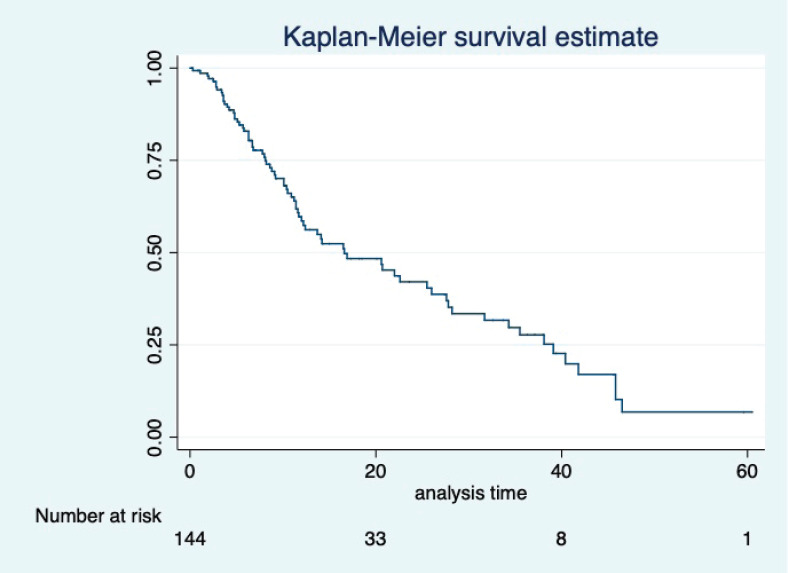
Kaplan Meier graph showing the PFS of the whole population.

**Figure 2.**
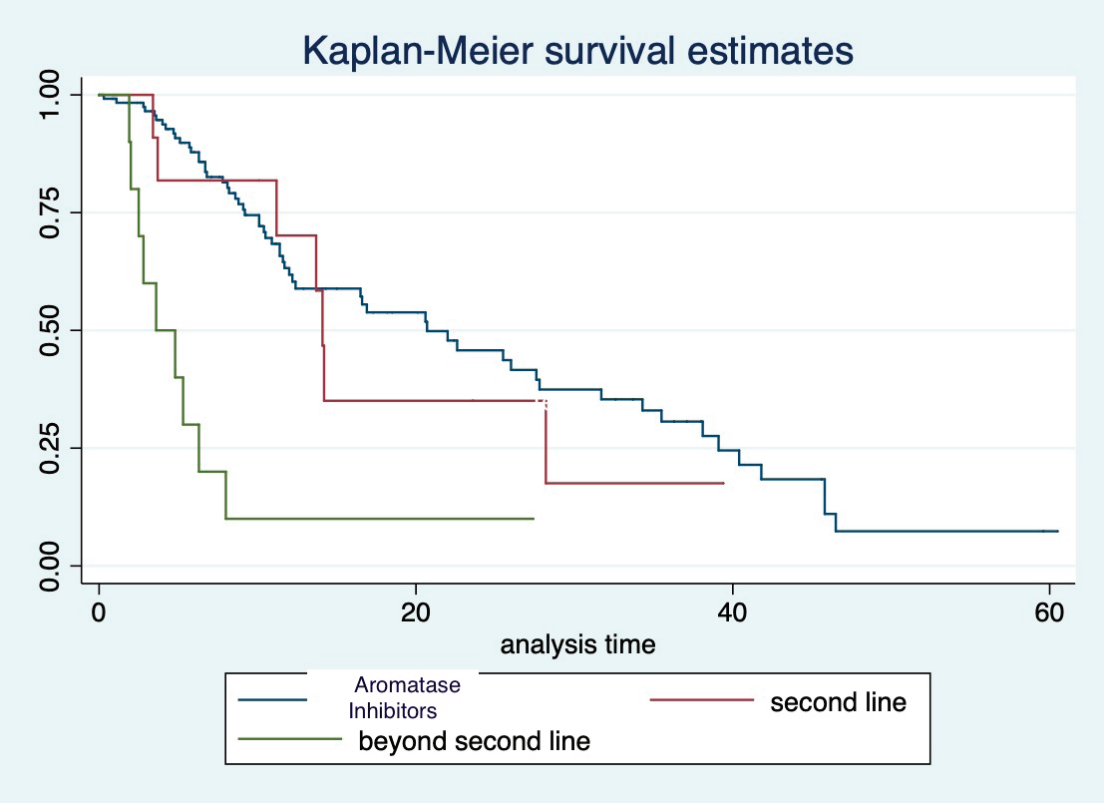
Kaplan Meier graph showing the PFS of the population treated with aromatase inhibitors (AI); Fulvestrant and beyond two lines of therapy.

**Figure 3.**
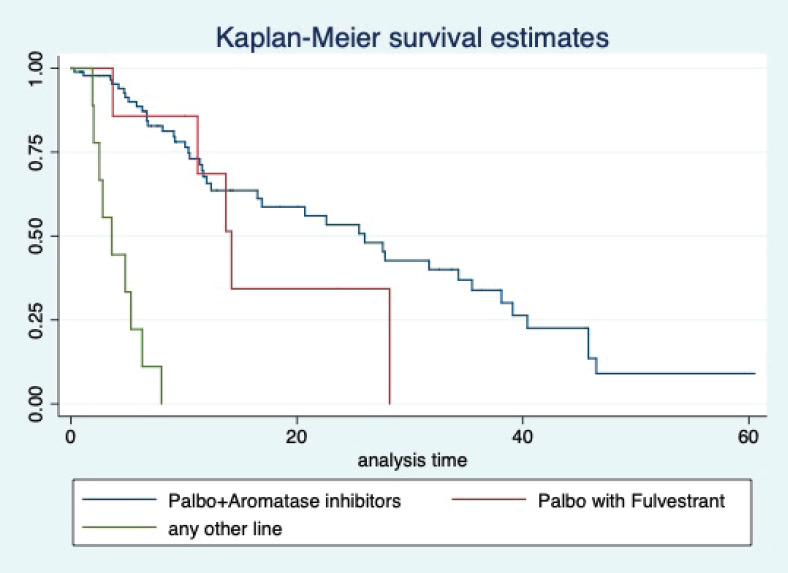
Kaplan Meier graph showing the PFS of the patients treated with palbociclib with aromatase inhibitors (AI); Palbociclib with fulvestrant and beyond two lines of therapy.

**Figure 4.**
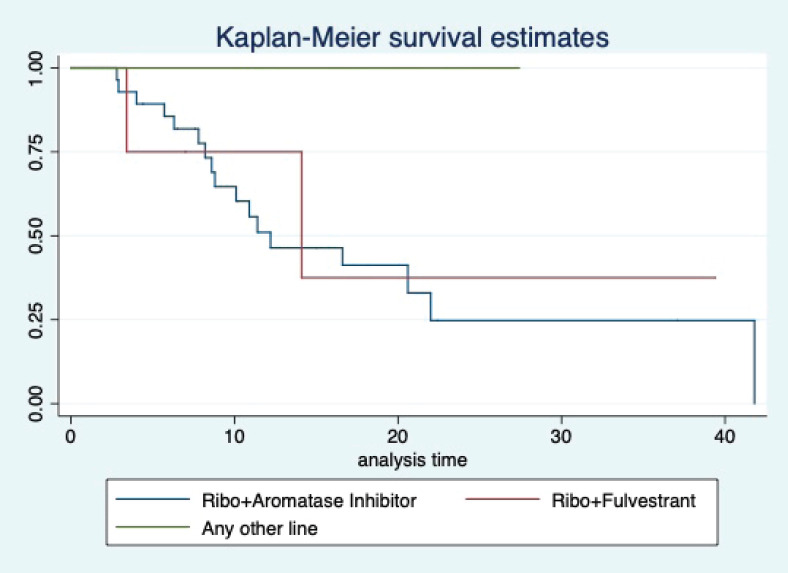
Kaplan Meier graph showing the PFS of the patients treated with ribociclib with aromatase inhibitors (AI); Ribociclib with fulvestrant and beyond two lines of therapy.

**Figure 5.**
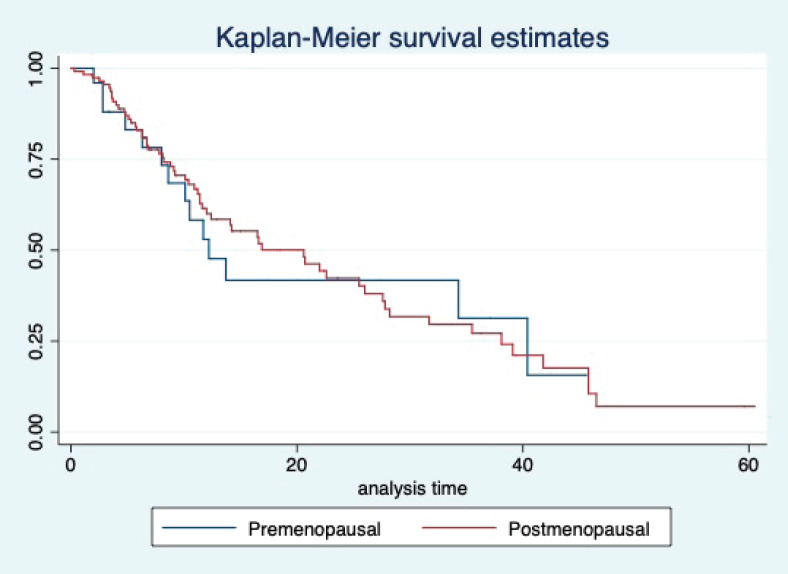
Kaplan Meier graph showing the PFS of the patients based on their menopausal status.

**Figure 6.**
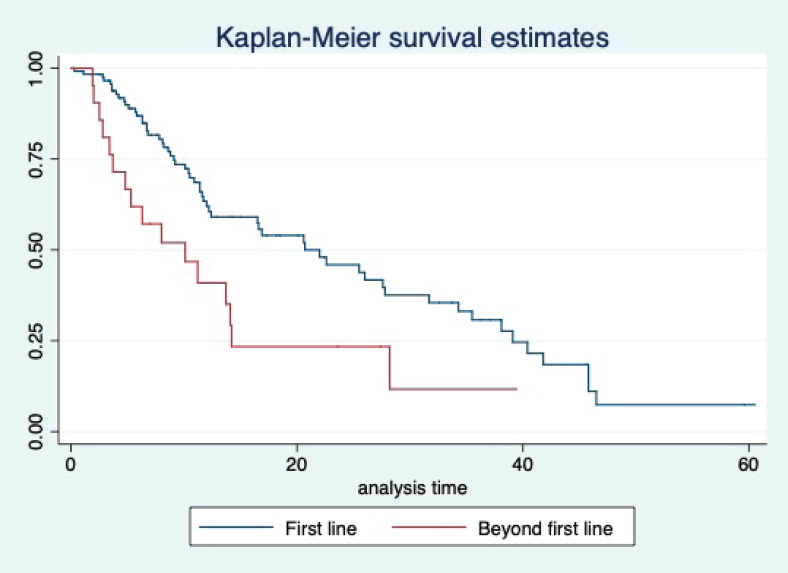
Kaplan Meier graph showing the PFS of the patients based on line of treatment.

At a median follow-up of 20.2 (95% confidence interval (95% CI): 13.5–28.3) months, the median PFS of the study population was 16.5 (95% CI: 11.6–25.5) months, as shown in Figure 1. When used with AI, the median PFS was 20.7 (95% CI: 12–27.8) months, and in combination with fulvestrant, the median PFS was 14.1 (95% CI: 3.7–NR) months as shown in Figure 2. The median PFS when the drug was used beyond two lines of treatment was only 3.6 (95% CI: 1.9–6.3) months, as shown in Figure 2. With palbociclib, the median PFS with AI was 26 (95% CI: 16.5–35) months, and with fulvestrant, it was 14.2 (95% CI: 3.7–NR) months, as shown in Figure 3. In contrast, with ribociclib, the median PFS with AI and fulvestrants was 12.2 (95% CI: 8.6–22) and 16.5 (95% CI: 3.9–NR) months, respectively, as shown in Figure 4. For premenopausal female, the median PFS was 12.2 (8–40.4) months and it was 20.6 (12–26) months for those who were postmenopausal as shown in Figure 5. Median PFS was 20.7 (12.2–27.8) months for those who received CDK4/6 inhibitors in the first line, and it was 10.1 (3.7–14.2) months who have received after prior systemic therapy as shown in Figure 6.

**Figure 7.**
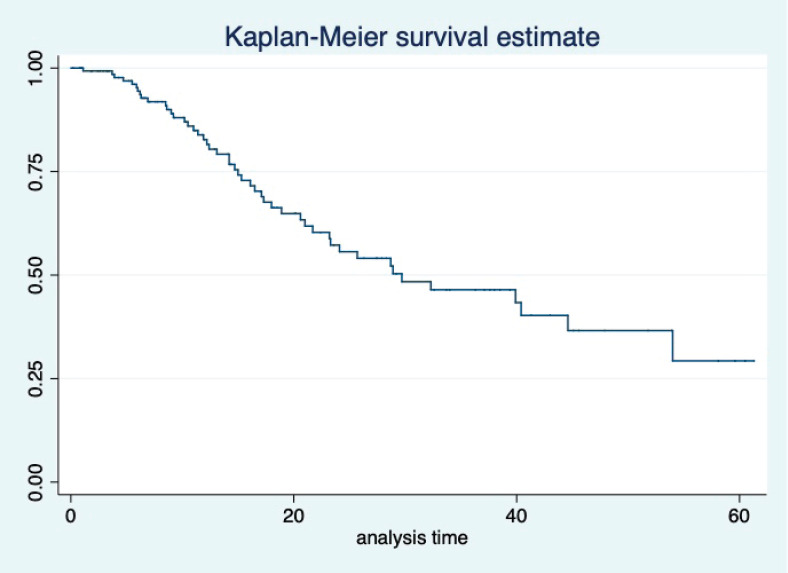
Kaplan Meier graph showing the OS of the whole population.

**Figure 8.**
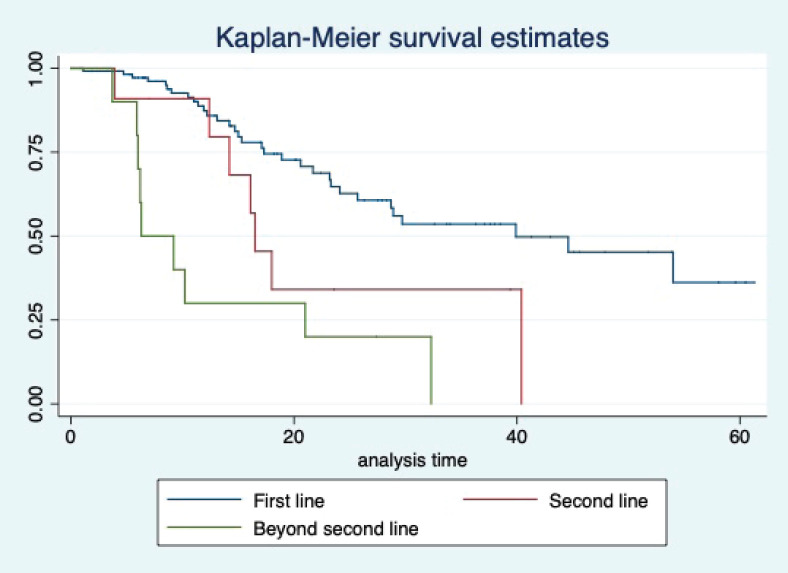
Kaplan Meier graph showing the OS of the population treated with aromatase inhibitors (AI); Fulvestrant and beyond two lines of therapy.

**Figure 9.**
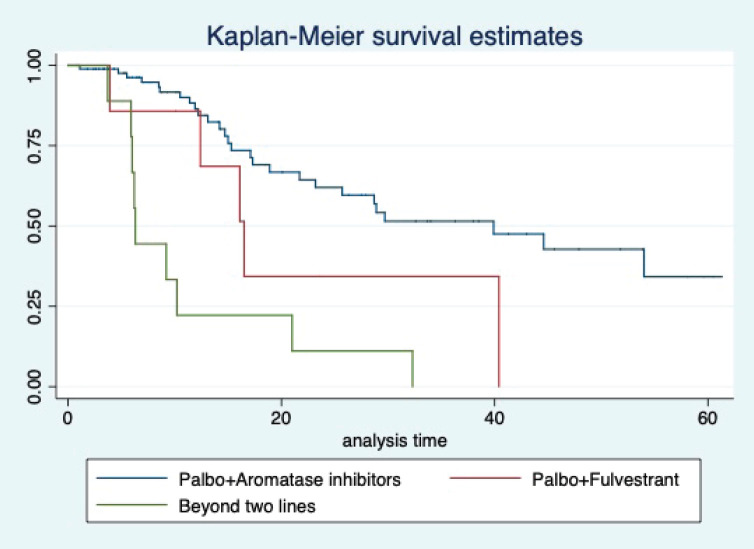
Kaplan Meier graph showing the OS of the patients treated with palbociclib with aromatase inhibitors (AI); Palbociclib with fulvestrant and beyond two lines of therapy.

**Figure 10.**
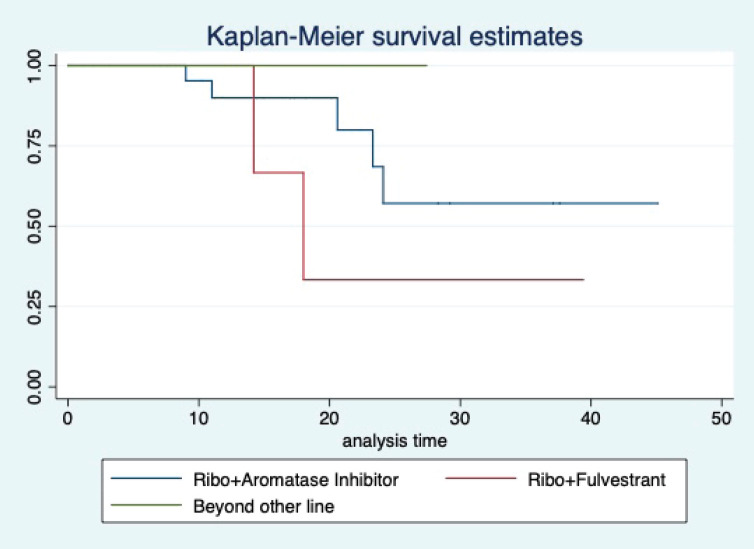
Kaplan Meier graph showing the OS of the patients treated with ribociclib with aromatase inhibitors (AI); Ribociclib with fulvestrant and beyond two lines of therapy.

**Figure 11.**
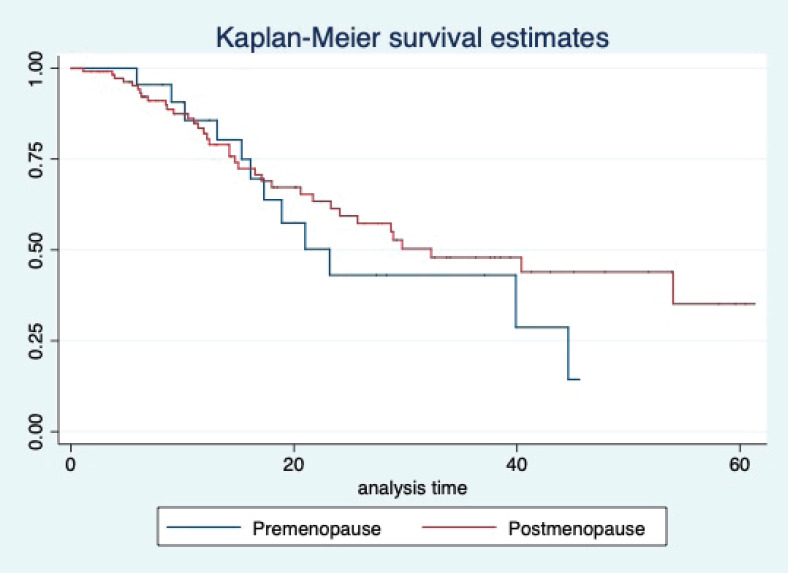
Kaplan Meier graph showing the OS of the patients based on their menopausal status.

**Figure 12.**
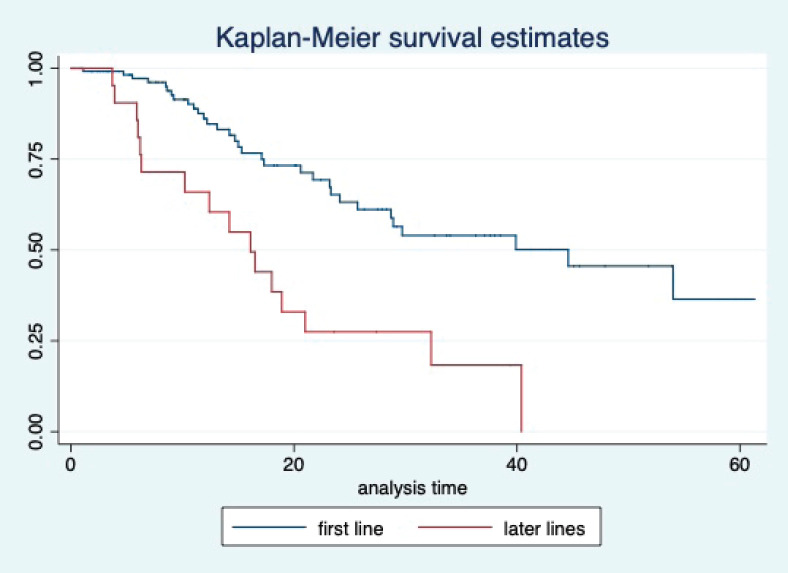
Kaplan Meier graph showing the OS of the patients based on the line of treatment

The median OS of the study population was 29.7 (95% CI: 21.7–44.6) months, as shown in Figure 7. When used with AI, the median OS was 39.9 (95% CI: 24.1–NR) months, and in combination with fulvestrant, the median OS was 16.5 (95% CI: 12.4–NR) months, as shown in Figure 8. With palbociclib, the median OS with AI was 39.9 (95% CI: 23.2–NR), and with fulvestrant, it was 16.1 (95% CI: 3.9–NR) months, as shown in Figure 9. In contrast, with ribociclib, the median OS with AI and fulvestrants was not reached (NR) (95% CI: 20.6–NR) and 18 (95% CI: 14.2–NR) months, respectively, as shown in Figure 10. For premenopausal female, the median OS was 23.2 (15.3–44.6) months and it was 32.3 (23.3–NR) months for those who were postmenopausal as shown in Figure 11. Median OS was 44.6 (24.2–NR) months for those who received CDK4/6 inhibitors in first line and it was 16.1 (6.3–21) months for who have received after prior systemic therapy as shown in Figure 12.

The Cox proportional model was used to determine prognostic factors in PFS and OS. For PFS, liver metastases, Pr score (<6) and line of therapy were significant in univariate analysis, and similar results were seen in multivariate analysis, as shown in Table 3. The presence of liver metastases, line of therapy and ER (<6) and Pr (<6) scores were significant in univariate analysis for OS. In multivariate analysis, liver metastases, Pr (<6) score and line of therapy were statistically significant, as shown in Table 4.

Neutropenia was the most common toxicity, as recorded in our series. Grade 3 or more neutropenia was seen in 52 (48%) patients. Febrile neutropenia was seen in 3 (2.1%) patients. Grade 3 anaemia was seen in 12 (9%) patients. Dose interruption and modification were done in 42 (30.6%) and 24 (22%) patients. We have permanently stopped the drug for only 3 (2.1%) patients when they have developed unacceptable toxicity. Those were one patient with grade 4 pulmonary toxicity, another one with liver toxicity in the form of deranged liver enzymes and the last one with recurrent anaemia in spite of adequate dose modification.

## Discussion

Most patients received CDK4/6 inhibitors as their initial treatment modality, mainly with aromatase inhibitors. The median PFS of the whole population was 16 months, and OS was 29.1 months. The median PFS with palbociclib was 20.2 months in the first line, while that with ribociclib was 12.2 months. The presence of liver metastases, prior therapy and Pr scores were poor prognostic markers for PFS. Neutropenia was the most common toxicity reported in our series.

The median age of our population was 53 years, comparable to what has been reported in other real-world studies from India [6, 7]. This reflects that breast cancer occurs at a younger age in India, with many presenting in an advanced stage. Only 27 (19%) patients were premenopausal at the time of presentation, which was similar to the IRIS study [8]. Ductal carcinoma was the most common histology in our study, similar to that reported by Rath *et al* [9]. Bone-only metastases were seen in 32 (23%) patients. In comparison, other studies have reported 17%–20% [6, 10].

The median PFS of the overall population was 16.1 months which was much more than that reported by Rath *et al* [9] where it was 7.7 months. This gross difference can be attributed by the fact that there were heavily pretreated patients in the study by Rath *et al* [9] compared to our research. However, when used in the first liner, the median PFS with CDK4/6 inhibitors was comparable in both studies and was around 20 months. Our study’s median PFS with first-line palbociclib was 26 months, similar to that reported in PALOMA 2 trial [11]. The median PFS with ribociclib with the first line in our study was 12.2 months. This PFS is much less than reported in landmark clinical trials [12]. This difference required further validation. With fulvestrant, the median PFS with both palbociclib and ribociclib was comparable to that reported in benchmark clinical trials [13, 14].

The median OS of our study population was 29.7 months, more than 27.1 months reported by Rath *et al* [9]. In the same study, the median OS in the first-line setting was not reached, while it was 39.9 months in our study [9]. These two differences can be attributed because the overall study population differed in both studies. Our study had more *de novo* patients than heavily pretreated patients in the other study.

In multivariate analysis, the presence of liver metastases, Pr score and line of therapy showed a prognostic factor for PFS and OS. Liver metastases and low Pr score did not show any prognostic significance in the pivotal clinical trials [11–14]. It requires further validation in other real-world studies. In another real-world study, prior treatment and performance score were found to be prognostic factors, and we have found prior treatment is a poor prognostic factor [9].

Neutropenia was the most common toxicity in our studies, similar to other studies. Dose interruption and modification were less compared to the landmark clinical trials due to inherent bias in underreporting of toxicities in retrospective studies. Permanent discontinuation of the therapy was done in 2.1% of the population, which is almost comparable to retrospective studies [7]. Incidence of febrile neutropenia was less than the study by Rath *et al* [9]. This difference is since, in the later study, heavily pretreated patients were more than ours in the later study [9].

Our study has some limitations. It is a retrospective study. The periodic assessments were not done at pre-specified time intervals.Uniform recordings of toxicities were not available in the available case records. Some patients have lost to follow-up, which has led to censoring. We have not considered the economic impact of treatment on the treatment outcome.

But our study has its strengths. We have tried to determine the efficacy of both palbociclib and ribociclib in a real-world setting. Almost all the patients received standard treatment as approved by the drug regulatory board of the country. Patients were given the drug through a compassionate access programme, thus reducing the cost of the treatment markedly.

## Conclusion

CDK4/6 inhibitors usage has led to improvement in PFS and OS in patients with HR+ MBC. The drugs are well tolerated. Ribociclib has not shown much improvement in PFS compared to palbociclib, and additional validation is required to look for a prognostic biomarker for this variation in response.

## Funding

No funding was obtained to conduct this study.

## Conflicts of interest

Sandip Ganguly – Institution has received primary investigator fees on his behalf from Novartis and AstraZeneca

Navonil Mukherjee – nothing to disclose

Sayan Mandal – nothing to disclose

Somnath Roy – Institution has received primary investigator fees on his behalf from AstraZeneca

Sanjit Agarwal – nothing to disclose

Bivas Biswas – Institution has received primary investigator fees on his behalf from AstraZeneca, Novartis, Roche, Pfizer

Joydeep Ghosh – Institution has received primary investigator fees on his behalf from AstraZeneca.

## Figures and Tables

**Table 1.** Baseline characteristics of the patients.

Variables	Total number (%)
Age 1. <50 years 2. ≥50	31 (21.5) 113 (78.5)
*De novo* disease 1. Yes 2. No	102 (71) 42 (29)
Site of metastases 1. Bone only 2. Visceral only 3. Bone + visceral	33 (23) 5 (3) 106 (74)
Histology 1. Ductal 2. Lobular	131 (91) 13 (9)
ER score 1. ≥6 2. <6 Pr score 1. ≥6 2. <6	133 (92) 11 (8) 92 (64) 52 (36)
Ki 67 1. <15% 2. 15%–30% 3. >30%	26 (18) 50 (35) 68 (47)
Grade 1 2 3	2 (1) 64 (44) 78 (55)

**Table 2.** Treatment details and response rate.

Parameters	Total number (%)
CDK4/6 inhibitors 1. Palbociclib 2. Ribociclib	108 (75) 36 (25)
Treatment regime 1. With AI+/− ovarian suppression 2. With fulvestrant 3. Beyond two lines	122 (85) 13 (9) 9 (6)
Response rate 1. Complete (metabolic) response 2. Partial response 3. Stable disease 4. Progressive disease 5. Not assessed	20 (16) 54 (44) 24 (20) 26 (21) 20 (19)
Overall response rate	74 (60)
Clinical benefit rate	98 (79)

**Table 3.** Univariate and multivariate analysis for PFS.

Variables	Category	PFS univariate			PFS multivariate		
		HR.	CI	*p*	HR	CI	*p*
Age (years)	<50	1					
	>50	1.17	0.68–2.02	0.563			
*De novo*	No	1					
	Yes	0.66	0.39–1.11	0.11			
PS	<2	1					
	≥2	1.08.	0.64–1.83	0.75			
Lung	No	1					
	Yes	1.23.	0.77–1.9	0.37			
Liver	No	1					
	Yes	2.39	1.4–4.05	0.01	2.09	1.22–3.58	0.007
Bone	No	1					
	Yes	0.88	0.52–1.4	0.65			
Pleura	No	1					
	Yes	0.85.	0.48–1.5	0.58			
Node	No	1					
	Yes	1.10	0.688–1.78	0.67			
ER score	Low	1					
	High	0.48	0.27–1.4	0.08			
Pr score	Low	1					
	High	0.65	0.48–0.87	0.05	0.66	0.49–0.9	0.009
Line		First	1				
	Beyond first	2.07	1.18–3.64.	0.011	2.07	1.18–3.66	0.011
Menopausal	Premenopausal	1					
status	Postmenopausal	0.9	0.5–1.63	0 75			

**Table 4.** Univariate and multivariate analysis for OS.

Variables	Category	PFS univariate			PFS multivariate		
		HR.	CI	*p*	HR	CI	*p*
Age (years)	<50	1					
	>50	0.75	0.49–1.31	0.37			
*De novo*	No	1					
	Yes	0.5	0.27–0.91	0.02			
PS	<2	1					
	≥2	0.76	0.3–1.47	0.42			
Lung	No	1					
	Yes	1.03.	0.58–1.82	0.9			
Liver	No	1			1		
	Yes	3.46	1.8–6.39	0.00	2.32	1.15–4.68	0.019
Bone	No	1					
	Yes	0.93	0.49–1.7	0.82			
Pleura	No	1					
	Yes	0.71.	0.34–1.47	0.37			
Node	No	1					
	Yes	1.03	0.57–1.87	0.9			
ER score	Low	1					
	High	0.29	0.12–0.71	0.007			
Pr score	Low	1					
	High	0.62	0.44–0.89	0.01	0.67	0.45–0.99	0.05
Line	First	1					
	Beyond first	3.15	1.18-3.64.	0.01	3.98	2.07-7.65	0.0003
Menopausal	Premenopausal	1					
status	Postmenopausal	0 .72	0 .37-1.40	0.3			
